# Hypovitaminosis D and prevalent asymptomatic vertebral fractures in Moroccan postmenopausal women

**DOI:** 10.1186/1472-6874-12-11

**Published:** 2012-04-24

**Authors:** Abdellah El Maghraoui, Zhor Ouzzif, Aziza Mounach, Asmaa Rezqi, Lahsen Achemlal, Ahmed Bezza, Saida Tellal, Mohamed Dehhaoui, Imad Ghozlani

**Affiliations:** 1 Rheumatology Department, Military Hospital Mohammed V, PO Box 1018, Rabat, Morocco; 2 Biochemistry Department, Military Hospital Mohammed V, Rabat, Morocco; 3 Statistics Department, Agronomic university Hassan II, Rabat, Morocco

**Keywords:** Osteoporosis, Vertebral fractures, VFA, DXA, 25(OH) vitamin D

## Abstract

**Background:**

Hypovitaminosis D is associated to accentuated bone loss. However, association between osteoporotic vertebral fractures (VFs) and vitamin D status has not been clearly established.

**Objective:**

To determine serum vitamin D status and to assess the association of vitamin D status with bone mineral density (BMD) and asymptomatic VFs prevalence using vertebral fracture assessment (VFA) in a cohort of Moroccan menopausal women.

**Methods:**

from June to September 2010, 178 menopausal women 50 years old and over were enrolled in this cross-sectional study. The mean ± SD (range) age, weight, height and BMI were 58.8 ± 8.2 (50 to 79) years, 73.2 ± 13.8 (35 to 119) Kgs, 1.56 ± 0.06 (1.43 – 1.79) m and 29.8 ± 5.9 (17.5 – 49.8) kg/m^2^, respectively. VFA images and scans of the lumbar spine and proximal femur were obtained using a GE Healthcare Lunar Prodigy densitometer. VFs were defined using a combination of Genant semiquantitative approach and morphometry. Serum levels of 25-hydroxyvitamin D (25(OH)D) were measured.

**Results:**

Among the 178 women, 45 (25.2%) had densitometric osteoporosis, and on VFA, VFs (grade 2 or 3) were detected in 20.2% while grade 1 were identified in 33.1%. The mean values of serum levels of 25(OH)D were 15.8 ± 11.6 ng/ml (range: 3.0 – 49.1) with 152 patients (85.3%) having levels <30 ng/ml (insufficiency) and 92 (51.6%) <10 ng/ml (deficiency). Stepwise regression analysis showed that presence of VFs was independently related to age, 25(OH)D and densitometric osteoporosis.

**Conclusion:**

our study shows that advanced age, hypovitaminosis D and osteoporosis are independent risk factors for asymptomatic VFs in Moroccan postmenopausal women.

## Background

Osteoporosis and low-trauma fractures in elderly people are a substantial and increasing burden of ill health worldwide and the rates of hip and other fractures are rapidly increasing in developing countries [1]. Vitamin D status has for long been recognized as an important factor for bone health and its contribution to fracture risk has recently received increased attention. It is demonstrated that hypovitaminosis D is associated to accentuated bone loss, leading to an increased risk of osteoporotic fractures. Moreover, new data suggest that its benefits extend beyond healthy bones [2,3].

Direct sunlight stimulates the production of vitamin D in the skin from 7-dehydrocholesterol. Other sources of vitamin D include some natural foods (e.g., fatty fish), fortified foods (e.g., margarine), and supplements. The amount of vitamin D produced through exposure to UVB radiation depends on skin type: the darker the skin, the more sunlight is required to produce a given amount of vitamin D. The duration of UVB irradiation needed to produce a certain quantity of vitamin D in a particular skin surface depends on season, time of day, and geographical location. The higher the latitude, the lower the UVB intensity, and the fewer months and hours per day during which vitamin D is produced.

Vitamin D insufficiency remains an under recognized problem in adults in the world [4]. In the United States, national population statistics from the 3 rd National Health and Nutrition Examination Survey (NHANES III) indicate that 41% of men and 53% of women have hypovitaminosis D [5]. Paradoxically, many recent studies have shown a high prevalence of vitamin D deficiency in many tropical and sunny countries such as Turkey [6], India [7], Iran [8], United Arab Emirates [9] and Saudi Arabia [10]. In Morocco, where sunlight is abundant, throughout the year, one study showed that during the summer season, vitamin D insufficiency is very common in healthy adult Moroccan women (91%) [11]. In this study, lack of sun exposure and veiled clothing style were the most important factors that influenced vitamin D status.

Many studies linked hypovitaminosis D and hip fractures. However, association between osteoporotic vertebral fractures (VFs) and vitamin D status has not been clearly established. In contrast to hip fractures, the majority of VFs are asymptomatic. Worldwide, underdiagnosis of VFs is a major problem. Additionally, studies have demonstrated that prevalent VFs can predict future VFs [12], hip fracture [13], and increased mortality during the following decade [14,15].

X-rays are the gold standard for identifying VFs. However, recent studies showed that vertebral fracture assessment (VFA) using dual-energy X-ray absorptiometry (DXA) machines has demonstrated great utility for vertebral visualization [16-18]. The combination of low radiation exposure, low cost, high technical reproducibility and easy data storage renders the method attractive providing a rapid and convenient way for clinicians to identify patients with VFs [19].

In view of limited data on vitamin D status and its association with bone mineral density (BMD) or VFs risk in Moroccan women, the objectives of this study were to determine serum vitamin D status and to assess the association of vitamin D status with BMD and asymptomatic VFs prevalence using VFA.

## Methods

### Subjects

This was a cross-sectional study conducted from June 2010 to September 2010 with menopausal women 50 years old and over, living in the region of Rabat, recrutized from the general population through advertisements and “word of mouth” in local hospitals. The city of Rabat is located on the western side of Morocco (latitude 34° and longitude 6°). Rabat is the capital of Morocco with a diverse population representing most Moroccans. Morocco has a population of 32,148,716 (estimation from the 2004 population Census), most of whom are Caucasians, and Rabat is a modern city of 620 996 inhabitants (51.6% female).

One hundred and seventy eight consecutive women who had no previous diagnosis of osteoporosis were entered into the study. General exclusion criteria were non-Caucasian origin and diseases, drugs, and other major determinants known to affect bone metabolism. Thus, we excluded subjects with gastrectomy, intestinal resection, recent thyroid dysfunction or hyperparathyroidism, recent severe immobilization or treatment with corticosteroids (more than 3 months) or any medication interfering with bone metabolism. Our institutional review board approved this study. The procedures of the study were in accordance with the Declaration of Helsinki, and formal ethics committee approval was obtained for the study. All the participants gave an informed and written consent. Each subject completed a standardized questionnaire designed to document putative risk factors of osteoporosis. History of fractures, lifestyle (alcohol consumption, gymnastics or jogging/walking, smoking) and diet (milk, yogurt, cheese) habits were also recorded. The women were asked whether they usually drank milk, coffee, or alcohol, if they ate cheese or yogurt, if they did gymnastics or jogging/walking, and if they smoked tobacco. Menstrual and reproductive history were assessed: all patients were menopausal since at least one year. Height and weight were measured in our centre before DXA measurement, in light indoor clothes without shoes. Body mass index (BMI)] was calculated by dividing weight in kilograms by height in meters squared.

### BMD measurement

Bone mineral density was determined by a Lunar Prodigy Vision DXA system (Lunar Corp., Madison, WI). The DXA scans were obtained by standard procedures supplied by the manufacturer for scanning and analysis. All BMD measurements were carried out by 2 experienced technicians. Daily quality control was carried out by measurement of a Lunar phantom. At the time of the study, phantom measurements showed stable results. The phantom precision expressed as the coefficient of variation percentage was 0.08. Moreover, reproducibility has been assessed in clinical practice and showed a smallest detectable difference of 0.04 g/cm^2^ (spine) and 0.02 (hips) [20,21]. Patient BMD was measured at the lumbar spine (anteroposterior projection at L1-L4) and at the femurs (i.e., femoral neck, trochanter, and total hip). The World Health Organization (WHO) classification system was applied, defining osteoporosis as T-score ≤ −2.5 and osteopenia as −2.5 < T-score < −1. Study participants were categorized by the lowest T-score of the L1–4 lumbar spine, femur neck, or total femur.

VFA was classified using a combination of Genant semiquantitative (SQ) approach and morphometry in the following manner: each VFA image was inspected visually by two clinicians (IG and AR who had a previous training session in VFA) to decide whether it contained a fracture in any of the visualized vertebrae. Each vertebra that was judged as fractured by visual inspection by any of the investigators was measured using built-in morphometry and assigned a grade based on Genant SQ scale [22], where grade 1 (mild) fracture is a reduction in vertebral height of 20-25%, grade 2 (moderate) a reduction of 26-40%, and grade 3 (severe) a reduction of over 40%.

### Laboratory evaluation

All blood samples were collected under fasting conditions on the same day of acquisition of the VFA images from June 2010 to September 2010 (a sunny period), between 8 and 10 a.m., stored at −20°C, and analyzed at the same time. The serum concentrations of calcium (adjusted for the albumin concentration), phosphorus, alkaline phosphatase, and creatinine were determined according to automated standard laboratory methods. The serum concentration of 25-hydroxivitamin D (25(OH)D) was measured using electrochimiluminescence on ELECSYS 2010 analyser (Roche Diagnostics, Mannheim).The intra and inter-assay variation coefficients in our laboratory were 10.5% and 17.8%, respectively. In the present study, 25(OH)D values of ≤ 30 ng/ml were defined as vitamin D insufficiency and of ≤ 10 ng/ml as vitamin D deficiency. When comparing to other studies in the literature, serum 25(OH)D concentrations presented in nanomole per liter or microgram per liter were transformed into nanogram per milliliter.

### Statistical analysis

Results are presented as means (SD) and categorical variables are expressed as frequencies. To compare patients with and without VFs, chi-square test and ANOVA were used firstly. Potential risk factors were entered to a stepwise conditional binary regression analysis and the resulted odds ratios with 95% confidence intervals were reported. Logistic-regression models were used to analyze the most important factors related to the presence of moderate/severe VFs. Only the variables significantly (p < 0.05) associated with moderate/severe VFs in the univariate analysis were included in the final logistic-regression. Because the colinearity between BMD sites, one after another was put in logistic-regression and the best model was selected. Hosmer and Lemeshow test were used to adjust the logistic-regression models. The level for significance was taken as p ≤ 0.05. Excel 2007 and SPSS 15.0 were used for statistical analysis.

## Results

In this cohort of 178 women, the mean ± SD (range) age, weight and BMI were 58.8 ± 8.2 (50 to 79) years, 73.2 ± 13.8 (35 to 119) Kgs and 29.8 ± 5.9 (17.5 – 49.8) kg/m^2^, respectively (Table 1). Thirty six women (20.2%) had a history of traumatic peripheral fracture in younger age (radius = 19, tibia = 16, femur = 1). The prevalence of osteoporosis (any site) was 25.2% (n = 45).

**Table 1:** Characteristics of the population study (n = 178)

	Mean ± SD	Range
Age (years)	58.8 ± 8.2	50–79
Weight (Kg)	73.2 ± 13.5	35–158
Height (m)	1.56 ± 0.06	1.43–1.79
BMI (Kg/m²)	30.0 ± 5.5	14.5–50.8
Number of parity	5.4 ± 2.6	0–13
Years since menopause	10.9 ± 9.5	1–30
BMD lumbar spine (g/cm²)	0.984 ± 0.1	0.550–1.410
BMD total hip (g/cm²)	0.870 ± 0.1	0.310–1.283
T-score lumbar spine (SD)	−1.3 ± 1.3	−4.9–2.1
T-score total hip (SD)	−1.2 ± 1.2	−4.7–2.1
25OH vitamin D (ng/ml)	15.8 ± 11.6	3.0–49.1

In these 178 women, 74% of vertebrae from T4–L4 and 82% from T8–L4 were adequately visualized on VFA. The percentage of vertebrae not visualized at T4, T5, and T6 levels was 72.6%, 56.6, and 35.4% respectively. Vertebral fractures (grade 2 or 3) were detected in 20.2% (36/178) of these women while grade 1 were identified in 33.1% (59/178). Fractures were most common in the mid-thoracic spine and at the thoraco-lumbar junction.

**Figure 1:**
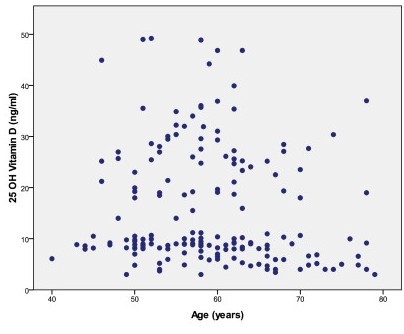
Vitamin D distribution in our population study (n = 178).

The serum concentrations of calcium (adjusted for the albumin concentration), phosphorus, alkaline phosphatase, and creatinine were within normal range. Serum levels of 25(OH)D distribution according to age is represented in Figure 1. The mean values were 15.8 ± 11.6 ng/ml (range: 3.0 –49.1). Globally, 152 patients (85.3%) had levels <30 ng/ml (which defined vitamin D insufficiency), 117 (65.7%) had levels < 20 ng/ml, and 92 (51.6%) had levels <10 ng/ml (which defined vitamin D deficiency).

As would be expected, the prevalence of VFA-detected fractures globally increased with age and as BMD declined. Comparison between patients without VFs and with grade 1 or grade 2/3 VFs showed that the latter were significantly older, lighter and had lower BMD and lower values of 25(OH)D in a significant linear association (Table 2). A significant linear association was also found between the severity of VFs and the prevalence of vitamin D insufficiency: 97% of grade 2/3 VFs patients were vitamin D insufficient compared to 85% of patients with grade 1 VFs and 78% of patients without VFs.

**Table 2:** Comparison between patients with and without vertebral fractures (VFs) according to the Genant semiquantitative classification

	Patients without VFsN = 81	Patients with grade 1 VFsN = 62	Patients withgrade 2 and 3 VFs N = 35	*p*
Age (years) : m (SD)	54.5 (5.7)	60.6 (7.9)	66.0 (7.7)	0.0001
Weight (Kg) : m (SD)	74.6 (12.6)	76.2 (14.5)	68.2 (12.3)	0.017
Height (m) : m (SD)	157.6 (5.9)	156.3 (5.9)	155.1 (6.3)	NS
BMI (Kg/m²): m (SD)	30.1 (5.7)	31.1 (5.4)	28.3 (5.0)	NS
History of fracture: n (%)	14 (17.3)	12 (19.4)	9 (25.7)	NS
Menopause duration (years) : m (SD)	6.8 (5.6)	12.1 (9.0)	18.4 (10.7)	0.0001
Number of parities: m (SD)	4.3 (2.3)	5.3 (2.1)	5.8 (2.8)	0.005
Vitamin D (ng/l) : m (SD)	18.7 (12.8)	17.5 (9.7)	6.2 (7.2)	0.0001
Vitamin D insufficiency: n(%)	63 (77.8)	56 (90.3)	35 (100)	0.001
Total hip BMD (g/cm²): m (SD)	0.910 (0.17)	0.792 (0.12)	0.786 (0.11)	0.001
T-score total hip (SD) : m (SD)	−0.8 (1.2)	−1.3 (1.2)	−1.9 (0.9)	0.0001
Lumbar spine BMD (g/cm²): m (SD)	1.131 (0.19)	0.980 (0.15)	0.912 (0.10)	0.007
T-score lumbar spine (SD) : m (SD)	−1.1 (1.2)	−1.4 (1.2)	−1.9 (0.8)	0.006

Comparison between patients according to vitamin D status (Table 3) showed that subjects with vitamin D insufficiency or deficiency had lower lumbar spine and hip T-scores and more prevalent VFs.

**Table 3:** Comparison of patients according to the vitamin D status (values in ng/ml)

	Patients with normal vitamin D(25(OH)D ≥ 30)N = 25	Patients with vitamin D insufficiency(10 ≤ 25(OH)D < 30)N = 60	Patients with vitamin D deficiency(25(OH)D < 10) N = 92	P
Age (years): m(SD)	58.5 (6.7)	58.1 (6.9)	59.4 (9.1)	NS
Weight (Kgs): m(SD)	78.7 (16.3)	71.9 (13.1)	73.8 (12.8)	NS
Lumbar spine BMD (g/cm^2^): m(SD)	1.030 (0.16)	0.956 (0.16)	0.982 (0.15)	NS
Total hip BMD (g/cm^2^): m(SD)	0.998 (0.18)	0.854 (0.14)	0.841 (0.14)	NS
Lumbar spine T-score: m(SD)	−0.8 (1.4)	−1.5 (1.2)	−1.3 (1.2)	0.0001
Total hip T-score : m(SD)	−0.2 (1.5)	−1.3 (1.1)	−1.4 (1.1)	0.0001
Prevalence of osteoporosis: n(%)	4 (16.0)	15 (25.0)	26 (28.3)	NS
Prevalence of VFs: n(%)	5 (20.0)	36 (60.0)	53 (57.6)	0.002
Prevalence of grade 2/3 VFs: n(%)	1 (4.0)	6 (10.0)	28 (30.4)	0.0001

Stepwise logistic regression analysis for the presence of grade 2/3 VFs (grade 1 VFs were excluded from this analysis) included age, weight, years of menopause, number of pregnancies, 25(OH)D, and the presence of osteoporosis any site (lumbar spine, femoral neck and/or total hip). It showed that presence of grade 2/3 VFs was independently related to age, serum levels of 25(OH)D and the presence of osteoporosis (Table 4). When patients with grade 1 VFs (VFs yes/no) were included in the analysis, the results were almost identical (data not shown).

**Table 4:** Multiple logistic regression analysis for the presence of grade 2/3 vertebral fractures

	Exp (B)[95% CI]	*p*
Age	1.303 [1.148–1.480]	<0.0001
25 OH Vitamin D	0.692 [0.525–0.913]	<0.0001
T-score ≤ −2.5 (any site)	4.860 [1.078–21.917]	0.04

## Discussion

We analyzed in this study the associations between serum 25(OH)D levels, BMD, and prevalence of VFs in a population of asymptomatic postmenopausal women. Our study demonstrated that increasing age, osteoporosis and hypovitaminosis D were the most important independent factors associated to the presence of prevalent VFs.

In agreement with our results, Gerdhem et al. evaluated 986 women and determined that patients with vitamin D levels below 20 ng/ml (50 nmol/L) had a twofold increased risk of presenting any type of osteoporotic fractures compared to women with levels above this value [23]. However, only symptomatic osteoporotic fractures were assessed in this study and it is well known that the majority of VFs is actually asymptomatic and mostly undiagnosed [24]. More recently and as shown in our study, Lopes et al. [25]. found similar results: vitamin D insufficiency, age, and femoral neck BMD were the most important factors for prevalent moderate/severe vertebral fractures in a series of 415 eldery Brazilian women assessed with VFA.

Evaluation of the relationship between 25(OH)D and hip fractures by several investigators has shown that low serum levels of serum 25(OH)D is a relevant risk factor for hip fractures [26,27]. In fact, 50% of women presenting hip osteoporotic fractures in the United States had serum vitamin D values lower than 12 ng/ml (30 nmol/L) [28]. Similar results were found among different populations which suggested that vitamin D serum concentrations could constitute a useful predictor of hip fracture risk in the elderly [29-31].

In contrast to our study, other authors evaluating the incidence of VFs observed no significant association with vitamin D insufficiency [32]. However, the authors failed to exclude women who had a history of VFs from the control group, which detracts from a definitive conclusion. A population-based cohort study by Garnero et al. [33]. showed that serum 25(OH)D was not associated with incident fracture in postmenopausal French women (mean age 62.2 years). Roddam et al. [34]., using a nested case–control design, showed no such association in a British study population (mean age 52.6 years). However, the mean age of the participants in these studies was low, and for younger patients vitamin D status may play a less important role in fracture risk. A study targeting postmenopausal Japanese-American women living in Hawaii who had high 25(OH)D concentrations (mean value, 32 ng/ml) showed no association between vitamin D status and fracture risk [35]. However, this study evaluated all types of fracture and relied upon X-ray films of asymptomatic patients to diagnose vertebral fracture; thus, it cannot be directly compared to our study.

Age and low BMD are independent risk factors for prevalent VFs. This evidence lends credence to the new recommendation by the ISCD that VFA should be utilized in all women with low BMD. We determined the best model of logistic-regression was with hip BMD and not with lumbar spine BMD, which may be due to established effects of aging on this site. It is well known that degenerative alterations that accompany ageing, such as osteophytes, and facet joint osteoarthritis and/or extraskeletal calcification around the lumbar spine may falsely increase the measurement of BMD at this site.

It is now well demonstrated that prevalent VFs are associated with an increase in morbidity and mortality. Moreover, post-menopausal women with severe VFs are at the highest risk of subsequent vertebral and nonvertebral fracture [36]. In fact, the severity of a previous fracture was found to be a better predictor of future nonvertebral fracture risk than BMD. In this context, the finding that low (<10 ng/mL) 25(OH)D concentrations are associated with a high prevalence of moderate/severe VFs (30% in our study) supports the inclusion of this modifiable risk factor as part of the screening, and the identification of postmenopausal women with a high risk of VFs.

The interpretation of a serum level of 25(OH)D in the “insufficient” range is challenging for several reasons. Although there are several ways to measure 25(OH)D (radioimmunoassays, enzyme-linked assays, and liquid chromatography with mass spectrometry), the precision and accuracy of the assays, especially in nonreference laboratories, remain problematic. In one study, identical serum samples were provided to 6 different laboratories, and the chemiluminescent assay tended to return higher values for 25(OH)D [37]. The 25OHD assay used in our study was the Roche electrochemiluminescence assay that has now been withdrawn from the market, in part because it underestimated 25(OH)D levels, especially at low levels and it only measures 25(OH)D3. Thus, one can speculate that the 25(OH)D reported levels are lower than the true levels, which may partly explain the very high proportion of women with 25(OH)D < 10 ng/ml. However, none of our patients was under D2 therapy and a cross-calibration data with another assay (Liaison, Diasorin) in 30 patients showed high correlation (r = 0.92; p < 0.0001) (data not shown). Moreover, the same observation of high prevalence of vitamin D deficiency had been reported in a previous study where the prevalence of values of 25(OH)D lower than 15 ng/mL were found in 43% of a series of young healthy Moroccan women during the summer season and using the technique of chemiluminescence (Liaison, Diasorin) [11]. In our study, the prevalence of extremely low vitamin D status (52%) is indeed quite striking. Symptomatic vitamin D deficiency is still a common problem in Morocco and cases of rickets and osteomalacia are still observed. However, the serum concentrations of calcium and phosphorus in our series were within the reference range and none of our patients had clinical evidence of osteomalacia.

Finally, 25(OH)D levels change with the seasons, exposure to sunlight, and dietary intake. For example, in northern latitudes, serum levels of 25(OH)D decline by 20% from late summer to midwinter, whereas 30 minutes of full-body exposure to the sun during the summer rapidly generates vitamin D. Rabat which is located at the 34th latitude is almost sunny through all the summer season. Cultural habits may be the explanation that limits sun exposure in many Moroccan women as well as low vitamin D intake. In our country, food is not supplemented with vitamin D. Hence, cutaneous synthesis would be the major source of the vitamin in ambulatory women of this age group. The influence of clothing style on 25(OH)D level, has been showed in a previous study where in the summer season and after a multilinear regression analysis, wearing a veil was found to be an independent predictor of hypovitaminosis D [38] and similar conclusions has been reported in Lebanon [39], Saudi Arabia [40] and Turkey [41].

Our study had strengths and limitations. The assessment of fracture was carefully conducted using standard procedures of acquisition and standard reading of all VFAs. All the morphometric assessments were made by two experienced investigators after training sessions and after a previous global visualization. Before diagnosis of fracture, a non-osteoporotic origin was considered for each deformity as vertebral deformities can be due to developmental abnormalities, Scheuermann’s disease sequelae and degenerative changes. Attention has been paid to osteoporotic depressions of the central end plates of the vertebrae, as osteoarthritic changes occur only on the anterior part of the vertebrae. However, even though history of trauma was inquired, we cannot exclude that some subjects did not report remote traumas. The main limitations lie in the cross-sectional nature of the study and in the procedures used to select subjects, who were all volunteers and ambulatory. The Rabat population may not be adequately representative of the whole population. However, since the population living in the area of Rabat is a balanced mixture of the various regions constitutive of the country, we believe the impact on prevalence estimate is limited. Another limitation is the dosage of PTH which could not be performed in this study. Indeed, in a population like this, with a so high prevalence of low levels of vitamin D, we would expect to have a secondary hyperparathyroidism, which, in turn, contributes to bone loss.

## Conclusion

Our study shows that the prevalence of vitamin D insufficiency is very high in Morocco and that hypovitaminosis D is an independent risk factor for asymptomatic VFs in Moroccan postmenopausal women as were advanced age and densitometric osteoporosis. These findings are consistent with the inclusion of the serum 25(OH)D dosage in the fracture risk evaluation and with the recently released report of the Institute of Medicine, which recommended that healthy adults take 600 IU daily to maintain skeletal health [42].

## Competing interest

The authors declare that they have no competing interest.

## Authors’ contribution

AEM designed the study, did the statistical analysis and wrote the paper. ZO participated in the study design, enrollment of subjects and performed the biological exams. AM participated in the data collection. AR participated in the data collection. LA participated in the data collection. AB participated in the data collection. ST performed the biological exams. MD participated in the statistical analysis. and IG participated in the study design, enrollment of subjects and participated in the statistical analysis. All authors read and approved the final manuscript

## Pre-publication history

The pre-publication history for this paper can be accessed here:

http://www.biomedcentral.com/1472-6874/12/11/prepub
